# Molecular Mechanisms of Inflammasome in Ischemic Stroke Pathogenesis

**DOI:** 10.3390/ph15101168

**Published:** 2022-09-21

**Authors:** Maria Grazia Puleo, Salvatore Miceli, Tiziana Di Chiara, Giuseppina Maria Pizzo, Vittoriano Della Corte, Irene Simonetta, Antonio Pinto, Antonino Tuttolomondo

**Affiliations:** Department of Health Promotion, Maternal and Infant Care, Internal Medicine and Medical Specialties, “G. D’Alessandro”, University of Palermo, Piazza delle Cliniche n.2, 90127 Palermo, Italy

**Keywords:** ischemic stroke, neuroinflammation, NLRP3 inflammasome, NLRP1 inflammasome, NLRP2 inflammasome, NLRC4, therapeutic strategies

## Abstract

Ischemic stroke (also called cerebral ischemia) is one of the leading causes of death and severe disability worldwide. NLR inflammasomes play a crucial role in sensing cell damage in response to a harmful stimuli and modulating the inflammatory response, promoting the release of pro-inflammatory cytokines such as IL-18 and IL-1β following ischemic injury. Therefore, a neuroprotective effect is achieved by inhibiting the expression, assembly, and secretion of inflammasomes, thus limiting the extent of brain detriment and neurological sequelae. This review aims to illustrate the molecular characteristics, expression levels, and assembly of NLRP3 (nucleotide-binding oligomerization domain-like receptor [NLR] family pyrin-domain-containing 3) inflammasome, the most studied in the literature, in order to discover promising therapeutic implications. In addition, we provide some information regarding the contribution of NLRP1, NLRP2, and NLRC4 inflammasomes to ischemic stroke pathogenesis, highlighting potential therapeutic strategies that require further study.

## 1. Introduction

Stroke has a notable social and health impact and is the third most common cause of death after ischemic heart disease and neoplasms, and the first cause of irreversible disability in the countries of the Western world [1]. Stroke can be divided into two subtypes according to pathogenetic characteristics, namely, ischemic and hemorrhagic. The first is the most frequent epidemiologically, occurring in 80–90% of cases. Several factors, such as the severity and location of the brain injury and the duration of the reduction in blood flow, affect the reversibility or permanence of neurological deficits that may affect the cognitive, motor, and sensorial spheres. Considering the physiopathogenetic mechanisms underlying ischemic stroke, it is possible to resort to two therapeutic expedients: reperfusion and neuroprotection. Reperfusion can occur by mechanical or pharmacological thrombolysis through the employment of antithrombotic, antiaggregant, or thrombolytic agents for the purpose of restoring blood flow in the damaged brain area [2]. Neuroprotection instead aims to reduce neuronal death by modulating different intra and extracellular signals implicated in the processes that cause cell damage.

Currently, the main objective of scientific research is to discover a new drug that can effectively trigger neuroprotection mechanisms, preserving that brain area which, after the ischemic damage occurs, is called the ischemic penumbra. In fact, in the context of an ischemic stroke, the inflammatory response that follows from brain damage, contrary to what happens in the case of infectious diseases or toxic noxae, does not have beneficial effects but perpetuates ischemic damage [3].

An inflammasome has been defined [4] by its sensor protein (a PRR), which oligomerizes to form a pro-caspase-1 activating platform in response to DAMPs or PAMPs. There are five members of PRRs that have been confirmed to form inflammasomes, namely, the nucleotide-binding oligomerization domain (NOD), leucine-rich repeat (LRR)-containing proteins (NLR) family members NLRP1, NLRP3, and NLRC4, as well as absent-in-melanoma 2 (AIM2) and pyrin.

An essential contribution to neuroinflammation is then made by signals, such as PAMPs (pathogen-associated molecular patterns) and DAMPs (damage-associated molecular patterns), that interact with specific receptors exposed on the extracellular surface called PRRs (pattern-recognition receptors) to provoke the activation of innate immunity [4].

The phenomenon of neuroinflammation is further exacerbated by the inflammasome, intracellular protein complexes associated with innate immunity, which, following activation, trigger cell death and pyroptosis via cleaved caspase-1, after conversion of the precursors of inflammatory cytokines IL-18 and IL-1β into mature forms [5].

In this review, we have focused more on the examination of NLRP3, the NLR (nucleotide-binding oligomerization domain (nod)-like receptor) family pyrin-domain-containing 3, in consideration of its high expression in the brain tissue and the pivotal role played in modulating the inflammatory response to ischemic injury [6]. In addition, we analyzed the different molecules capable of inhibiting or blocking NLRP3 inflammasome signaling; thus, representing potential and innovative therapeutic strategies.

Finally, we reviewed other inflammasomes such as NLRP1, NLRP2, and NLRC4, the functions of which need to be clarified in the context of pathogenetic mechanisms that aggravate the sequelae of ischemic stroke. Gaining additional knowledge paves the way for discovery of new molecular targets capable of interfering with the neuroinflammation determined by the inflammasomes above-named.

## 2. An Overview of the Pathogenesis of Cerebral Ischemia

From a physiopathological perspective, there are three main mechanisms responsible for cerebral ischemia, namely, endothelial dysfunction, atherosclerosis, and neuroinflammation [7].

Following a stroke, an “ischemic cascade” is triggered and characterized by different cellular and molecular mechanisms such as inflammation and apoptosis, oxidative stress, and excitotoxicity with sometimes irreversible damage to endothelial cells, glia, and neurons [8].

Mediators that come into play due to an ischemic cerebral insult differ depending on the neuroinflammation stage. In the initial phase, adhesion molecules, proteases, prostanoids, TNF-α, IL1α, IL1β, CXCL7, CCL5, CXCL4, CX3CL1, and leukotrienes, are involved. NADPH oxidase, CXCL8, iNOS, COX-2, and some cytokines such as IL-1, IL-10, TNF-α, IL-6, IL-20, and IL-17 are involved in the process’s progress. Some cytokines, such as TGF-β, IL-17, IL-10, and IL-23 [7], mediate the term of neuroinflammation.

Unlike glia and endothelial cells, neurons are more prone to metabolic dysfunction and cell death because they are more sensitive to hypoxia conditions that occur during cerebral ischemia [9].

Insufficient oxygen and glucose supply causes oxidative phosphorylation failure and impedes ATP synthesis [10]. Cell ATP deficiency causes dysfunction of the Na^+^/K^+^ ATPase pump, and such electrolyte imbalance is responsible for extensive anoxic depolarization in glial cells and neurons [10]. As a result of such depolarization, voltage-gated calcium channels open at the pre-synaptic terminal. Hence, in the intracellular environment, a significant calcium concentration increase occurs. This event induces the release of the primary excitatory neurotransmitter, glutamate, into the synaptic cleft [11,12]. Therefore, glutamate resorption damages not only neurons but also astrocytes [13]. In addition, the accumulation of calcium within the mitochondria determines the genesis of reactive oxygen species (ROS) [14], the opening of the permeability transition pore responsible for mitochondrial depolarization [15], and finally neuronal death.

ROS in glial cells and neurons determine the activation of MAPKs and NF-kB pathways involved in caspase-mediated apoptosis. In addition, ROS can lead to mitochondrial and endoplasmic reticulum disruption, facilitating the release of cytochrome c (such as a pro-apoptotic protein) and additional Ca^2^ ions in the cytosol. This phenomenon determines ischemic insult amplification and apoptosis [16,17].

Concurrently, the increment of the intracellular concentration of sodium also induces water inflow through the osmosis mechanism and consequently cytotoxic cerebral oedema [10,18].

The hypoxemia consequential to ischemia is also responsible for anaerobic glycolysis with increased lactate and subsequent acidosis with a pro-oxidant action. The increase in hydrogen ions stimulates the conversion of O^−2^ (anionic super-oxide) into hydrogen peroxide (H_2_O_2_) and the hydroperoxyl radical (OH^−^) [6,19].

As a result of ischemic brain damage, both the production of ROS, damaged tissues, and necrotic cells determine the trigger of an inflammatory response mediated by cells of innate immunity [20,21]. In addition, astrocytes, microglia, neurons, and endothelial cells release cytokines that secrete pro-inflammatory cytokines, such as tumour necrosis factor-alpha (TNF-α), interleukin-1 (IL-1), IL-6, and IL-18, accountable for neuronal and glial cell death following ischemic stroke [22,23]. The above-mentioned pro-inflammatory cytokines could activate the production of adhesion molecules such as intercellular adhesion molecule-1 (ICAM-1), vascular adhesion molecules (VCAMs), and selectins (e.g., P-selectin, E-selectin), on the endothelial cells, platelets, leukocytes [24,25].

The first cells to be activated following Ischemic brain damage are those of microglia. Subsequently, the monocytes/macrophages and the neutrophils also reach the ischemic area passing the blood–brain barrier (BBB) interacting with the adhesion molecules [26,27]. During reperfusion, glial cells and neurons secrete chemo-attractant protein-1 monocyte (MCP-1/CCL2), which causes the migration of neutrophils into the infarcted area [28].

The brain damage resulting from the reperfusion is also exacerbated by the release of additional pro-inflammatory cytokines (i.e., TNF, IL-1, IL-6, IL-12, and IL-18) and matrix metalloproteinases (MMPs, mainly MMP-2 and MMP-9) by natural killer cells, macrophages and neutrophils [23,28]. In addition, these cytotoxic agents activate inducible pathways NO synthase (iNOS) and cyclooxygenase-2 (COX-2), which impair endothelial cells [29]. Following ischemic stroke, the expression of MMPs is responsible for disrupting the BBB, aggravating cerebral oedema, hemorrhagic transformation, and, ultimately, death of neuronal and glial cells [30].

The death of neuronal cells triggers the activation of TLRs expressed by oligodendrocytes, astrocytes, and microglia. These receptors represent a type of PRRs, crucial pattern recognition receptors (PRR) capable of recognizing different PAMPs and DAMPs such as HMGB1 (High-mobility group box 1), hyaluronic acid, other thermal shock proteins (HSP), and mRNA [31,32].

PRRs include intracellular PRRs (nod-like receptors (NLRs), NOD1, and NOD2), DC-specific ICAM3-grabbing non-integrin (DC-SIGN), 3-receptor complement (CR3), C-type lectin receptors (CLR), Dectin 1, or CLEC7A (C-type lectin domain family 7 member A), Dectin 2, or CLEC6A (C-type lectin domain family 6 member A), myeloid DAP-12-associated lectin (MDL-1) and trigger receptors expressed on myeloid cells (TREM-1) [33].

In addition, all perturbations of the cellular microenvironment are intercepted by specific sensors capable of mounting an inflammatory response such as inflammasomes. These include NLRC4, AIM2, NLRP1, NLRP2, NLRP3, NLRP6, NLRP7, NLRP12, and Pyrin inflammasome [25].

Following a cerebral ischemic injury, the stimulation of PRR by DAMPs causes the activation of NF-κB and MAPK signaling pathways and the subsequent increase in the production of pro-inflammatory cytokines, thanks to the involvement of inflammasomes [34].

The pathways NF-κB and MAPK partially induce the expression and activation of NLRP1 and NLRP3 inflammatory proteins in vivo and in vitro under ischemic conditions [34]. Reports show that ischemic stroke increases the expression and activation of NLRP3 inflammatory proteins in glial cells and neurons [17].

## 3. NLRP3 Inflammasome

### 3.1. NLRP3 Inflammasome: Molecular Characteristics

During transient or permanent vascular obstruction, neurons, vascular cells, and glial cells are exposed to toxic stimuli responsible for cell damage. Dangerous molecular signals such as pathogen-associated molecular patterns (PAMPs) and damage-associated molecular patterns (DAMPs) determine the activation of innate immunity through a panoply of molecules, including cytokines and inflammasomes, which intensify neuroinflammation and ischemic detriment. [35]. The involvement of inflammasome in inflammatory processes was suggested for its capability to act as a complex activator of caspase-1 through the release of IL-1β following the cleavage of its preform. Scientific research has highlighted two types of inflammasomes, i.e., NLR inflammasomes [5] and PYHIN inflammasomes (an acronym for Pyrin and HIN domain-containing protein). Among these, most studies focused on NLRP3 inflammasome, also called NALP3 or cryopyrin, encoded by CIAS-1 (also namely cold-induced autoinflammatory syndrome 1). It is expressed both by cells of the immune system and nervous system and implicated in phlogistic response which takes place in immune, infectious, and inflammatory pathologies [25].

The NLRP3 inflammasome is a threefold protein that plays a pivotal role in the innate immune system due to its capability to respond to bacterial, viral, and mycotic infections and to preserve cell homeostasis from other aseptic toxic stimuli [36]. It is composed of PYD (an amino-terminal pyrin domain), the NACHT domain (a central nucleotide-binding and oligomerization domain, also known as NOD), and LRR (a C-terminal leucine-rich repeat domain). The early stage of the assemblage of the inflammasome is characterized by the interaction between the pyrin domain of NLRP3 and the pyrin domain of ASC (a caspase-recruitment domain), which regulates cell death and inflammatory response engaging with pro-caspase-1. Subsequently, through the bond with CARD (caspase recruitment domain), the activation of the inflammasome is obtained.

The oligomerization of NLRP3 after activation requires energy, so the ATPase activity of NOD is essential for the assembly of inflammasome [37,38]. The role played by the LRR domain is still unclear because it is involved in ligand sensing, but novel research has shown that it does not take part in inflammasome activation or autoinhibition [39].

Once the oligomerization of NLRP3 in response to noxious stimuli occurs, inflammasome NLRP3 is generated by assembling the cytoplasmic proteins NLRP3, the precursor of caspase-1 and ASC [40]. The latter interacts with NLRP3 through the Pyd domain and with the precursor of caspase-1 through homotypic CARD/CARD interactions. Subsequently, activated caspase-1 contributes to the induction of cellular pyroptosis with the maturation and secretion of cytokines IL-18 and IL-1β after cleavage of the precursors of the pro-inflammatory forms. Mature forms of the cytokines mentioned above stimulate the enrollment of different immune system cells, including neutrophils [41].

As many research studies have shown, the role of NLRP3 inflammation in exasperating and perpetuating neuroinflammation following an ischemic stroke is well established [42].

### 3.2. Role of NLRP3 in Cerebral Ischemia

The NLRP3 inflammasome is a critical component of the innate immune system that mediates caspase-1 activation and the secretion of pro-inflammatory cytokines IL-1β/IL-18 in response to microbial infection and cellular damage [43]. It is implicated in ischemic damage at the renal, myocardial, hepatic, and cerebral levels. The maturation and increase of IL-1β and IL-18 cytokines, that play a pivotal role in neuroinflammation, depend on activating the NLRP3/caspase-1 axis [25]. Furthermore, caspase 1 is capable of inducing both apoptosis and pyroptosis, a particular type of cell death [44].

Pyroptosis is mediated exclusively by the activated form of caspase-1. In detail, caspase-1 plays a pivotal role in the pyroptosis process as it cleaves off the amino-terminal gasdermin-N from the carboxy-terminal gasdermin-C domains in gasdermin-D [45]. Caspase-1 is responsible for excessive secretion of proinflammatory cytokines and chemokines following the destruction of the plasma membrane. These cytokines and chemokines drive the migration of leukocytes and other immune system cells into the infarcted area, amplifying brain tissue damage and causing neuronal death [46]. An increased expression of the NLRP3 inflammasome and caspase-1, ASC, IL-18, and IL-1β are detected in neuronal cells. The inflammatory role of the inflammasome is further supported by the neuroprotection obtained by suppressing its activity by therapeutic administration of anti-caspase 1 and intravenous immunoglobulin [43]. In 1986, Koizumi et al. [47] experimented on murine models in which a transient occlusion of the middle cerebral artery (tMCAO) was induced and silenced the NLRP3 gene, demonstrating how, when inhibiting the expression of the inflammasome mentioned above following ischemic damage, the inflammation and consequently also the size of the infarct and the volume of the oedema were reduced, and the permeability of the blood brain barrier (BBB) was not impaired.

Caspase-1, an inflammasome-activated NLRP3 enzyme capable of lysing other proteins, is also implicated in the pathophysiology of ischemic stroke. In fact, following a cerebral infarction, increases in the level of expression of caspase-1 in both astrocytes and neuronal cells occurs. In contrast, upon knockout of the gene of caspase-1 in transgenic mice, the inflammatory response is inhibited after a provoked experimental cerebral ischemia. Consequently, the cerebral damage is reduced [12]. This hypothesis is further endorsed by a study that demonstrated in rat hippocampal slices, where oxygen/glucose deprivation was previously induced, a therapeutic role played by molecules capable of inhibiting caspase-1 [48]. Therefore, studies in the literature support the hypothesis that caspase-1 plays a crucial role in the pathogenesis of ischemic stroke.

NLRP3 by means the active caspase-1 fragments elicit the maturation and secretion of pro-inflammatory cytokines IL-1β and IL-18, which belong to the IL-1β family and mediate subsequent immune responses. This “cytokine storm” fuels the inflammatory response to ischemic insult. On microglial cells, neurons, astrocytes, and endothelial cells are located cytokine receptors that can interact with such molecules to induce the expression of numerous inflammation-associated genes [25].

IL-1β destroys the BBB and consequently facilitates the infiltration of immune system cells in the central nervous system [49]. It also determines an increased expression of chemokines and stimulates the migration of leukocytes into the cerebral parenchyma. IL-18 also causes an augmented chemokine and adhesion molecules production in T-helper 1 (Th1), B cells, and NK (natural killer) cells [50]. In addition, the cytokine above-mentioned induces the expression of Fas ligand in glial cells, thereby exacerbating neuroinflammation and neuronal death [42]. In microglia cells, IL-18 results in increased expression of MMPs and caspase-1 [51].

The release, in large concentrations, of IL-1β promotes phosphorylation and subsequent activation of NMDA (*N*-methyl d-aspartate) receptor; it is responsible for excessive calcium flow which determines excitatory toxicity [24].

### 3.3. Expression of NLRP3 Inflammasome in Ischemic Stroke

Recent studies have revealed that the inflammasome complex is also constitutively expressed at the central nervous system level, unlike IL-18 and IL-1β [35]. Immunohistochemical analyses of infarcted brain tissue compared to healthy patients show higher expression levels for caspase-1 [52].

In brain tissue, astrocytes do not express NLRP3, ASC, and caspase-1. At the same time, significant expression levels have been found mainly in microglia, which therefore play a crucial role in activating inflammasome under pathological conditions of neuroinflammation [53]. The activation of microglia in a pathological context is not surprising, being cells that are part of the innate cerebral immune system. Moreover, in the case of ischemic brain damage, blood vessels are responsible for exacerbating damage due to the upregulation of NLRP3 inflammasome in both microglia and endothelial cells [54].

The proteins that make up the NLRP3 complex are expressed in the presence of an ischemic stroke by brain endothelial cells, but not in neuronal cells, as demonstrated by a study conducted by Yang et al. [55]. Instead, another study has shown their expression also in neurons of the primary cortex [56]. Brain endothelial cells constitute the interface between the central nervous system and the immune system and, consequently, represent a fundamental component of the NVU (neurovascular unit). This acronym refers to the neurovascular unit, a complex structure formed by several cells that interact with each other such as pericytes, neurons, glial cells, and cells of the endothelium. Nagyöszi et al. demonstrated that the NLRP3 inflammasome could be activated in the cerebral endothelium in a MAPK-dependent manner with subsequent release of the active IL-1β cytokine [57].

Currently, accumulating evidence has highlighted the essential role of the activation of signaling pathway of the inflammasome in the pathogenesis of ischemic stroke. Nevertheless, the pattern of protein expression forming the NLRP3 inflammasome complex in the different cytotypes of the central nervous system under pathological conditions of brain damage is still debated. Furthermore, differences in expression could be ascribable to the duration of the ischemic event, the interventions implemented and the diversity of models.

### 3.4. Activation of NLRP3 Inflammasome Pathway in Ischemic Stroke

Numerous scientific studies have shown that two complementary signals associated with cell damage are responsible for the assembly and activation of the inflammasome. However, for greater clarity, it would be desirable to define the characteristics and ways these specific signals contribute to inflammasome activation.

The constitutional expression levels of NLRP3 inflammasome in astrocytes, microglia, and neurons are relatively low in physiological conditions. Therefore, for inflammasome to be activated in response to a stressful stimulus for the cell, it is necessary to upregulate the expression of the proteins that make up the complex. This is possible thanks to a priming (or first) signal [36]. The first signal involves the interplay between DAMPs, alarmins, and cell debris released from necrotic cells of damaged parenchyma, and TLRs, IL-1R (I-1 receptor 1), NLRs (such as NOD1 and NOD2), and RAGE (receptor for advanced glycation products). This interaction determines the activation of NF-kB, a transcription factor [36]. NF-κB migrates into the cell’s nucleus, where it ties up DNA and initiates the transcription and translation of the precursor of IL-18 and IL-1β and of NLRP3 [58]. In the NF-κB signaling pathway, two signaling molecules such as MyD88 (Myeloid differentiation primary response 88) and TRIF (TIR-domain-containing adapter-inducing interferon-β) are implicated in the induction of NLRP3 inflammasome and pro-ILβ in response to ligands interacting with TLR4 [21]. In the MyD88-dependent pathway, MyD88, an adaptor molecule, binds to the cytoplasmic side of TLR4 with subsequent recruitment of IRAK-4 and IRAK-1 through death domains. After forming this bond with MyD88, IRAK-1 undergoes phosphorylation thanks to the activation of IRAK-4 and attaches to TRAF6 (TNFR- associated factor 6). A complex composed of MyD88, IRAKs, and TRAF6 is generated and known as myddosome. Following phosphorylation, IRAK-1 and TRAF-6 in turn activate TAK-1. TAK-1, activated by TRAF6 ubiquitination, activates the IκB kinase (IKK) complex consisting of the subunits α, β, and γ (also called NEMO) [59]. TAK1 then phosphorylates IKK-β and modulates the activation of NF-κB. Indeed, the phosphorylation and degradation of IκB allow the release of NF-κB that migrates into the nucleus. TAK-1 also activates MAPK (P38 and JNK) that phosphorylates AP-1 and CREB, allowing homodimerization and translocation in the nucleus with subsequent modulation of the inflammatory response.

In the MyD88-independent (or TRIF-TRAM dependent pathway), following TLR4′s link with the ligand, TRAM and TRIM associate with the TLR4, acting as a bridge for the link between TLR4 and TRIF. TRIF then interacts with TRAF6 by activating TAK1 with mechanisms similar to those observed in the MyD88-dependent pathway [58].

TLR signaling activation is responsible for the modulation of the transcription of the NLRP3 inflammasome but also in the post-transcriptional phase. Indeed, it affects its activation through the processes of phosphorylation and deubiquitination. The latter are always part of the priming phase [60].

NLRP3 is ubiquitinated in LRR domain and evidence has shown that a JAMM domain-containing Zn^2+^ metalloprotease called BRCC3 (BRCC36 in humans) stimulates NLRP3 deubiquitination and activation [61]. Studies have demonstrated that MAPKKK (mitogen-activated protein kinase kinase), TAK1 (TGF-β 1 activated kinase), and other TLR-signaling kinases(s) are capable of phosphorylation of NLRP3 or other inflammasome components, allowing rapid priming of NLRP3. In addition, recent findings have highlighted how the transcription factor IRF-1, activated by priming signals downstream of TLR4, stimulates the production of mtDNA (mitochondrial DNA), needful for activating the NLRP3 inflammasome [62].

In the induction process during the priming phase, FADD and caspase 8 are also involved, as recent publications have shown. They are both apoptotic signaling molecules, but they play the role of induction regardless of their apoptotic properties [21]. Caspase-8 activates NF-κB by interacting with the IKK complex, while FADD can play a double role as it can activate or repress the NF-κB, stimulating apoptosis [63].

Although it has been established that the priming signal plays a transcriptional role in activating inflammasome upregulating NLRP3 and IL-1b, new findings have also highlighted transcription-independent roles. For example, one study showed that acute priming with LPS may enhance NLRP3 without prior induction [64]. This rapid priming independent transcription involves IRAK-1 being phosphorylated by LPS independently of the IKK complex. This breakthrough supports the hypothesis that downstream NF-κB is not indispensable for promoting inflammasome activation via IRAK.

Various stimuli such as lysosomal membrane destabilization and consequent secretion of cathepsin, potassium efflux, release into the mitochondrial environment of mitochondrial ROS, increased intracellular calcium concentration, and acidosis may play a key role as a second signal necessary for the activation of NLRP3 [25]. In the ischemic area, necrotic cells secrete hydrogen ions that bind to ASIC1a, cause an influx of Ca^2+^ ions within the cytoplasmic environment, and activate the NLRP3 receptor. The latter can also be activated under hypoxia conditions by lactate production and its consequential acidosis due to anaerobic glycolysis. In addition, the secretion of cathepsin in the cytoplasm, due to the destabilization of the lysosomal membrane, may determine the activation of the NLRP3 receptor. Necrotic cells in the ischemic area can then release ATP that interacts with P2X4 and P2X7 receptors on astrocytes, neurons, and microglia [36]. This bond causes an outflow of potassium ions and a consequent reduction in the intracellular concentration of potassium. In conditions of ischemia in the cells a depletion of ATP occurs, resulting in a malfunction of the Na^+^/K^+^-ATPase pump. As a result of the pump malfunction, sodium concentration within the cell will increase, promoting intracellular water movement for osmosis and cellular oedema, further activating stimuli for the NLRP3 receptor. In addition, the electron transport chain in the mitochondria also fails, resulting in a relationship between ROS and TXNIP, which activates the NLRP3 receptor [65]. For the genesis of ROS and the increase of intracellular calcium, PKR (protein kinase R) is also activated, which activates the NLRP3 receptor.

The second signal elicits the activation of NLRP3 and the phosphorylation of ASC, favouring the assembly in the complex with NLRP3 and procaspase-1. The activated complex cleaves procaspase-1 to caspase-1. The latter molecule catalyzes the conversion reaction of proIL-1β and proIL-18 into IL-1β and IL-18, triggering a metabolic pathway that leads to pyroptosis and the amplification of neuroinflammatory damage [66].

**Figure 1:**
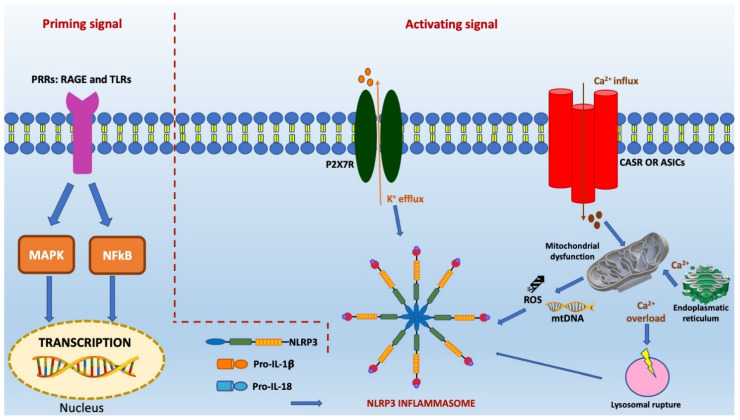
This figure illustrates the two steps of activation of the NLRP3 inflammasome signaling pathway in ischemic stroke, i.e., the priming and activating steps. In the first step, the elicitation of PRRs receptors, such as RAGE and TLRs, by endogenous cytokines and other ligands induces the activation of nuclear transcription factors, such as MAPK and NF-κB, and subsequent upregulation of NLPR3 levels and precursors of inflammatory cytokines. Following the priming step, an adaptor protein called ASC is assembled to the NLRP3, generating NLRP3 inflammasome complex. Its activation is favoured by the efflux of K^+^, the mobilization of Ca^2+^, lysosomal rupture, and mitochondrial dysfunction characterized by the formation of mtROS and mtDNA.

The mechanisms described above are crucial in determining the evolution of ischemic damage and also overlap with the two-stage activation of NLRP3 inflammasome. They are illustrated in Figure 1.

#### 3.4.1. K^+^ Efflux and Ischemic Neuronal Damaged Mediated by Inflammation

Among all the extracellular and intracellular factors that represent a stimulus capable of activating inflammasome, one of the most studied is K^+^ efflux.

After binding to ATP, the non-selective K^+^ cation channel is activated, leading to a cytosolic depletion of K^+^. This activates downstream signals that trigger the maturation and secretion of IL-1β [67]. The role of the outflow of potassium in the activation of the inflammasome has been further corroborated by several studies. They have shown that the reduction of the intracellular concentration of potassium is essential for the activation of the inflammasome pathway compared to other known stimuli. Among the receptors associated with the outflow of potassium capable of activating the inflammasome and releasing pro-inflammatory cytokines amplifying the ischemic damage, one of the most studied is P2X7R (P2X purinoceptor 7). It is verified through experiments conducted on mouse models [36].

Several studies have demonstrated the existence of inflammasome activation pathways independent of potassium output current and chemical compounds such as imiquimod, GB111-NH2, and CL097 capable of activating NLRP3 in the absence of K^+^ efflux [68]. In addition, an experiment on murine macrophages characterized by NLRP3R258W, a mutant form of NLRP3, highlighted how the activation of inflammasome occurs as a result of stimulation of LPS without intracytoplasmic potassium reduction. This research demonstrates that such an outflow is a sufficient but not indispensable condition in the triggering of the inflammasome [69].

However, further studies should be conducted to determine how the variation in the cytosolic concentration of potassium may cause conformational changes in inflammasome and regulate its activation.

#### 3.4.2. Role of Mitochondrial Dysfunction and ROS in Inflammasome-Related Neuronal Damage

Factors that play an essential role in activating inflammasome NLRP3 include damage to mitochondria, cytoplasmic organelles characterized by a double-membrane bound and the ability to produce both energy and ROS (reactive oxygen species) [70].

In response to NLRP3 stimuli, some mitochondrial molecules, such as MAVS (also known as mitochondrial antiviral-signaling protein), mitofusin 2, and cardiolipin, associate with NLRP3. For example, some studies show how MAVS interacts and determines inflammasome activation when induced by soluble stimuli such as nigericin, poly (I:C), and ATP. This does not happen with particulate matter such as alum, silica, and MSU [71]. In addition, other publications in the literature suggest that MAVS contributes to the activation of NLRP3 if induced by viral infectious agents. In fact, it recruits TRAF3 that, by ubiquitination of ASC, promotes its oligomerization and, therefore, the activation of inflammasome. These reactions do not take place for non-viral NLRP3 stimuli [72].

Mitochondria are also implicated in the activation of the inflammasome by ROS production. Indeed, following mitochondrial respiratory chain inhibition, mtROS (also known as mitochondrial ROS) are generated. They are capable of activating inflammasome in response to LPS and ATP. The physiopathological mechanism by which ROS modulate the assembly, through oligomerization, of NLRP3 inflammasome implies the prior induction of ROS-scavenging protein thioredoxin resolving from thioredoxin-interacting/inhibiting protein (TXNIP) and a cell-type-specific modulation [73,74]. Therefore, the release of mtROS and the subsequent activation of the inflammasome is inhibited by another free radical such as nitric oxide.

In addition to mitochondria, another possible source of ROS has long been thought to be lysosomal NADPH oxidase [75]. However, its role is now considered controversial because, as seen in a study conducted by Ma et al. [76], the deletion of NOX2, also called superoxide-generating NADPH oxidase 2, causes the diminution of the expression of NLRP3 in a traumatic brain lesion model, but not at the endothelium of the umbilical vein. These findings indicate that cytosolic ROS play a tissue-specific role in activating inflammasome.

In addition, NOX 4 (NADPH oxidase 4) by regulating CPT1A (carnitine palmitoyltransferase 1A) also elicits the activation of the inflammasome, enhancing the oxidation of fatty acids [77].

Mitochondrial DNA oxidation is considered another requirement for inflammasome activation; although more recent evidence has refuted the above hypothesis considering that both mtROS and mitochondrial dysfunction do not appear to be indispensable in determining the phlogistic activation of inflammasome [78].

#### 3.4.3. Ca^2+^ Mobilization in Inflammasome-Related Neuronal Damage

As shown by recent evidence, calcium also has a role in the activation of inflammasome. An increase in intracellular concentration of this ion in conjunction with reduced levels of potassium activates the inflammasome pathway and causes the release of IL-18 and IL-1β through plasma membrane-resident Ca^2+^ channels as TRPV2, TRPM2, TRPM7, and P2X7R (86). In addition, this role is further confirmed by the fact that suppressing the outflow of calcium through membrane receptors and preventing its overload in the endoplasmic reticulum through nigericin, ATP, and alum [79], inflammasome activation and L-1β maturation are inhibited. Through the ASICs (acid-sensing ion channels), plasma membrane-resident Ca^2+^ channels, calcium entry is allowed from the extracellular environment inside the cell [80].

The intracellular calcium increase is also determined by CASr (calcium-sensing receptor), which activates the phospholipase C pathway, thus favouring the assembly of inflammasome NLRP3 [81]. The modulation of calcium flow from the endoplasmic reticulum to the mitochondria takes place through crosstalk modulated by the Mitochondria associated membranes (MAM). It determines the activation of IP3R (inositol 1,4,5-trisphosphate receptor) and VDAC (mitochondrial voltage-dependent anion channels) [82]. Specifically, following the stimulation of GPRC6A receptors and calcium-sensing receptor (CASr), both G-protein-coupled receptors (GPCRS), phospholipase C is activated. It is responsible for the hydrolysis of PIP2 (4,5-bis-phosphatidyl phosphate), which becomes IP3, the acronym of inositol 1,4,5-triphosphate. Subsequently, the latter interacting with IP3R, an ionic ligand-gated channel, causes an intracytosolic efflux of calcium ions from the lumen of the endoplasmic reticulum (ER). When 2APB (2-aminoethoxy diphenylborinate) interacts with the IP3 receptor, both calcium efflux and inflammasome activation are prevented [83].

In addition, the intracellular increase of calcium activates inflammasome by two mechanisms, such as by increased production of mtROS with consensual destabilization of mitochondrial organelles and breakdown of lysosomes, releasing various molecules in the cytosolic environment, including cathepsin B, also activated by TAK1 kinase. As a result of the activation of MAPKs, cathepsin B determines the trigger of the first step of activation of inflammasome [75].

#### 3.4.4. Lysosomal Detriment in Inflammasome-Related Neuronal Damage

Mitochondria are not the only cellular organelles to be involved in the process of inflammasome activation. For example, after exposure and phagocytosis of particulate matter such as silica, cholesterol crystals, alum, amyloid-β, MSU, calcium crystals, and asbestos, lysosomes undergo a rupture resulting in dispersion of the contents in the cytosol and activation of NLRP3 provoked by a lysosomal-damaging agent (lysosomotropic) [64]. However, the metabolic pathways triggered by lysosomal rupture responsible for the inflammasome activation have yet to be clarified.

Lysosomal acidification could play a pivotal role, as suggested by a study that showed that H^+^ ATPase inhibitor bafilomycin A impedes activation stimulated by particulate matter [84]. This finding is further justified by the fact that the MSU can activate the inflammasome as it is responsible for the lysosomal acid environment leading to a massive propagation of Na^+^, resulting in an increased influx of water and cellular osmolarity and a reduction of the concentration of K^+^ inside the cell [85].

Current evidence claims that cathepsin B, a molecule released by lysosomes, contributes to the activation of inflammasome as it is necessary for the release of IL-1β. Furthermore, it was found that inflammasome is not activated in macrophages as a result of particulate exposure if they are treated with a chemical inhibitor for cathepsin B called CA-074-Me [84].

The inhibition of the activation of inflammasome NLRP3 by the aforementioned molecule could nevertheless represent an off-target effect as no differences were found between the activation of inflammasome in mice with cathepsin B deficit compared to those wild-types. This discovery is likely ascribable to the existence in the macrophages, which present cathepsin-B deficiency of other cathepsins such as C, L, S, and X; all of them are united by a redundant contribution in activating the inflammasome as a result of exposure to particulate matter. Cathepsin X is distinguished for its peculiar property of crucial action in nigericin-induced cell death [86].

Numerous studies in the literature endorse the hypothesis that cathepsin B is involved in the activation of the inflammasome, otherwise it is desirable that new studies furthermore clarify other mechanisms by which lysosomal detriment determines the activation inflammasome NLRP3.

#### 3.4.5. Non-Canonical Inflammasome Pathway and Alternative Inflammasome Pathway

**Figure 2:**
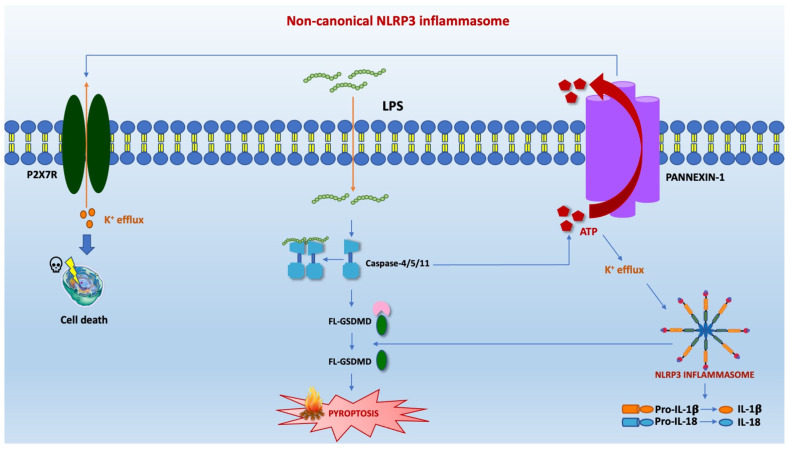
The non-canonical inflammasome pathway activates an endotoxic shock by stimulating LPS independently of TLR4 signaling. Caspase-11 plays a pivotal role in mice and caspases 4/5 in humans in this metabolic pathway. They can recognize and bind directly pent-acylated and hexa-acylated lipid A of the LPS without the mediation of TLR4. Subsequently, the caspases-4/5/11 provoke the cleavage of GSDMD, starting the process of pyroptosis. This phenomenon determines by caspase 11, the activation of pannexin-1, a channel protein that produces ATP. The release of ATP promotes the efflux of potassium. It is responsible for assembling NLRP3 inflammasome and the maturation and release of pro-inflammatory cytokines.

Two other modalities of activation of inflammasome besides the canonical one have been described in the literature, namely, the non-canonical one and the alternative one. The form of non-canonical activation of inflammasome constitutes a further defensive expedient against those pathogens that have evolutionarily acquired the ability to bypass cell surface TLR4 [87].The first activation mode resulted from the internalization of lipopolysaccharide (LPS) within the cytosolic environment through infection or transfection. The non-canonical way implicates the involvement, instead of Caspase 1, of Caspase 4-5-11. The caspases mentioned above intercept LPS by binding it directly and independently of TLR4, thanks to recognising pent-acylated and hexa-acylated lipid A [88]. A study conducted on murine elements with low levels of caspase-11 under basal conditions showed that the priming phase amplifies the inflammatory response [88]. However, priming was not essential in human cells expressing high caspase-4 [89]. In order to activate caspase 11 in macrophages, TLR4-dependent and TRIF-dependent IFN-α/β production, also involved in the modulation of the expression of pro-caspase 11, is essential. Caspase-4/5/11 are responsible for the pyroptosis process by cleavage of Gasdermin D (GSDMD) and its conversion into NT and CT fragments [87]. This phenomenon subsequently causes the activation of pannexin-1 which releases ATP. Subsequently, ATP activates an ATP-gated selective cation channel called the P2X7 receptor (P2X7R) [90], responsible for K^+^ efflux. The output current of potassium determines the assembly and activation of the inflammasome and the secretion of IL-1β (Figure 2).

**Figure 3:**
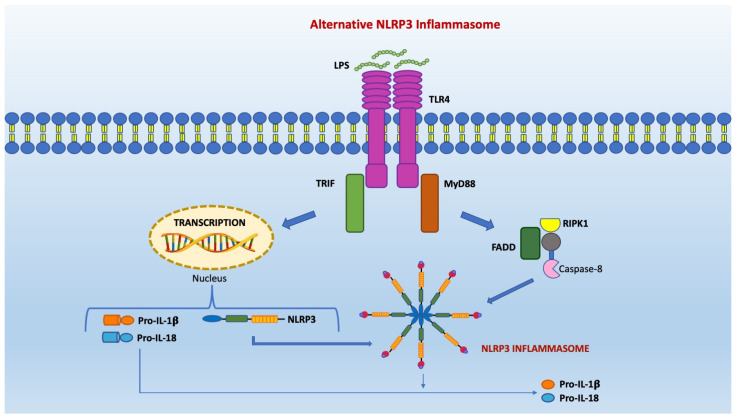
The alternative inflammasome pathway occurs in murine dendritic cells and in vivo in human monocytes. It involves TLR4-TRIF-RIPK1-FADD-CASP8 signaling and provokes the activation of NLRP3 inflammasome and the release of the mature form of the inflammatory cytokine IL-1β. Unlike in the canonical activation of the inflammasome, this process does not require potassium efflux, the participation of P2X7, or other stimuli secondary to LPS exposure.

The alternative pathway of inflammasome activation has been observed in human monocytes in response to LPS stimulation without the necessity for secondary stimulation. However, RIPK1 (short for receptor-interacting serine/threonine-protein kinase 1), FADD, and caspase-8 are required for inflammasome to become active. It is an independent potassium pathway that does not cause pyroptosis (Figure 3).

### 3.5. Direct and Indirect Inhibitors Targeting the NLRP3 Inflammasome Pathway for Ischemic Stroke Treatment

It is now established by several scientific works that inflammasome has a pivotal role in the pathogenesis of ischemic stroke. In consideration of the aforementioned assertion, it is easy to see how it is desirable to identify new therapeutic strategies that allow targeting upstream or downstream of the NLRP3 inflammasome pathway [25]. Furthermore, several molecules have been discovered to modulate the expression, assembly, activation, and secretion of the inflammasome. Therefore, inflammasome NLRP3 complex proteins, core proteins implicated in MAPK and NF-κB pathways, IL-1β, and IL-18 with their respective receptors and plasma membrane channels or receptors are counted among those molecules that can potentially serve as therapeutic targets for the pharmacological treatment of stroke [43]. In order to inhibit the activity of NLRP3 inflammasome, three crucial points have been identified. The first is the targeting of the ATPase activity of NLRP3 in order to prevent the formation of the inflammasome. The second involves a direct inhibition of NLRP3 that prevents the process of oligomerization. The third is aimed at blocking the activity of caspase-1 and consequently inhibiting the conversion of interleukin-1 to its mature form [91]. Furthermore, we have schematized the main inhibitors of the NLRP3 inflammasome pathway in Table 1.

#### 3.5.1. Small Molecules, Inflammasome Targeting, and Ischemic Neuroprotection

An experiment conducted by Fann et al. highlighted the neuroprotective role of SB 203580 (a P38 MAPK inhibitor), Bay 11 7082 (an NF-κB inhibitor), U 0126 (an ERK inhibitor), and JNK Inhibitor V (a JNK inhibitor) as these molecules in tMCAO murine models inhibit the activation of the inflammasome. Thereby, they prevent the resultant release of pro-inflammatory cytokines (IL-18 and IL-1β) and lead to a reduction in levels of NLRP3 inflammasome complex [34].

A neuroprotective effect is also carried out by glyburide and MCC950, which inhibit the oligomerization of NLRP3 and, in this way, determine a reduction of neuronal apoptosis and neurological dysfunction also containing the volume of infarction. In addition, glyburide exerts an effective anti-oedema action and limits oxidative stress, as shown by a study led by Peng et al., which opposed OGD-induced activity stress. At the same time, MCC950 exerts an anti-inflammatory activity [63,93,94].

MCC950, also called CP-456,773, can prevent both canonical and non-canonical activation of inflammasome NLRP3 already in nanomolecular concentrations, making it a selective NLRP3 inhibitor with very high efficacy. In fact, following an ischemic injury, it modulates the expression of Bax and cleaved caspase-3, increasing it and that of Bcl-2 in BV2, reducing it in the cells exposed to thrombin. In this way, it reduces neuronal cell apoptosis, limiting the size of ischemic injury and the extent of cognitive decay [93].

Another molecule that modulates the pathway of NLRP3 inflammasome is BHB, an acronym for beta-hydroxybutyrate, a ketonic metabolite that in monocytes interferes with NLRP3-induced ASC oligomerization, thus preventing the release of pro-inflammatory cytokines IL-18 and IL-1β [25]. In fasting conditions, caloric restriction, low-carbohydrate ketogenic diet, and high-intensity exercise, an increase in BHB production has been observed. This endogenous ketone can act as an alternative energy source for both heart and brain tissue. Therefore, it is necessary to define more deeply the relationship between ketones and inflammasomes in conditions of energy deficit [94]. Current knowledge supports the hypothesis that in inflammasome activation, BHB, unlike MCC950, has the ability to block the outflow of potassium from macrophages. In addition, MCC950 prevents both canonical and non-canonical inflammasome activation, while BHB exclusively modulates the canonical pathway of activation.

Recent studies have shown that, similarly to glyburide, other molecules are capable of selectively inhibiting the NLRP3 inflammasome pathway, such as Cy-09 [95]. However, it remains to be seen whether they can exert therapeutic effects in the case of ischemic stroke.

#### 3.5.2. NRF2 as Possible Therapeutic Target in Ischemic Neuroprotection

Nuclear factor erythroid-2 related factor 2 (Nrf2) is a redox-sensitive transcription factor that upregulates the expression of detoxification enzymes and antioxidants, piloting an antioxidant cellular response against numerous pathogenic noxae [96]. Redox signaling not only opposes the activation of inflammasome NLRP3, reducing the secretion of IL-1β but inhibits the synthesis of ROS through mediator Nrf2. Enhancing the Nrf2 antioxidant pathway through Isoliquiritigenin has been seen to reduce early brain damage following a cerebral haemorrhage caused by inhibiting the NF-kB or ROS-mediated activation of inflammasome NLRP3. Therefore, the employment of Nrf2 could be profitable from the therapeutic point of view as, by suppressing the activation of NLRP3 inflammasome through the TXNIP complex, the extent of brain damage secondary to ischemia and reperfusion is relieved [97].

#### 3.5.3. Nitric Oxide (NO) as Possible Therapeutic Target in Ischemic Neuroprotection

Numerous studies conducted on both myeloid cells and mouse and human models have pointed out that nitric oxide is capable of inhibiting the oligomerization of ASC pyroptosome, caspase-1 activation, and IL-1β secretion. Thereby, it impedes the assemblage and the successive activation of the inflammasome [98]. However, following cerebral ischemia, NO signaling pathways are downregulated, and, consequently, it is not possible to benefit from the advantages resulting from one of its most important properties, i.e., its anti-inflammatory property. Therefore, by regulating the pathways involving nitric oxide, it is possible to improve cerebral perfusion to implement a neuroprotective effect which may ameliorate prognosis [99]. Further research is needed to exploit nitric oxide as a potentially beneficial therapeutic agent.

#### 3.5.4. IFN as Possible Therapeutic Target in Ischemic Neuroprotection

Macrophages and dendritic cells are cells of the immune system that defend our body from harmful agents by producing interferons such as IFN-α and IFN-β [25].

It is ascertained that combating neuroinflammation resulting from ischemic damage improves the outcome and possibilities of neurofunctional rehabilitation. IFN-β could be a useful therapeutic expedient, a hypothesis supported by the advantages observed in patients with multiple Sclerosis disease undergoing systemic treatment with recombinant (r) IFN-β. This interferon exerts a neuroprotective action as it modulates the permeability of the blood–brain barrier (BBB), stabilizing endothelial tight junctions, hindering chemotaxis in the CNS through the downregulation of adhesion molecules and the reduction of expression of MMPs by leukocytes [100].

It also attenuates the proliferation of T lymphocytes and stimulates a shift of the latter from T helper (Th)1 to Th2.

With the aim of activating the interferon receptor called IFNAR, a member of the TLR family consisting of two subunits IFNAR1 and IFNAR2, several proteins that affect NLRP3 inflammasome and the subsequent release of pro-inflammatory cytokines need to be involved. Unlike other molecules capable of inhibiting the NLRP3 inflammasome pathway, many studies in the literature document the effects of the use of IFN-α and IFN-β on NLRP3 and other inflammasomes in numerous inflammatory diseases [101].

As demonstrated by some experiments conducted on bone marrow-derived macrophages of murine models, the IFN-1β could alleviate the production of IL-1β through the phosphorylation of STAT1 transcription factor. The latter suppresses the inflammasome NLRP3 and the increased production of IL-10, which in turn, through a mechanism dependent on STAT3 signaling, decreases the release of pro-IL-1α and pro-IL-1β. These trials emphasize the efficaciousness of IFN as a drug capable of blocking the activity of NLRP3 inflammasome and, consequently, counteracting neuroinflammation. However, it is desirable that further studies are conducted to explore better the mechanisms through which interferon suppresses NLRP3 inflammasome.

#### 3.5.5. Micro-NAs Modulators of Inflammasome

The NLRP3 inflammasome pathway is also modulated by long non-coding RNA XLOc_000647 [102] and numerous microRNAs such as mir 132 [103], and mir 22 [104]. The term miRNAs (microRNAs) refers to 22 nucleotides (nt) non-protein-coding RNAs capable of targeting mRNAs to inhibit translation, degradation, or cleavage. In so doing, they play a pivotal regulatory role in both plants and animals [25]. For example, recent studies have documented how mir-223 may represent a new therapeutic expedient in case of ischemic stroke as it binds to a conserved site in the 3 UTR of the NLRP3, inactivates the latter, and inhibits the release of IL-1β. This mechanism of action determines a significant reduction of cerebral oedema and a significant improvement of brain functions [105]. Furthermore, Mir-9 may also alleviate neuroinflammation related to atherosclerotic damage by inhibiting the JAK1/STAT1 metabolic pathway signaling pathway [106]. In addition, Mirna-133a-1, Mirna-377, and Mirna-155 also appear to have an impact on inflammasome activation [107].

Therefore, it might be promising to develop new therapeutic strategies to affect the inflammasome pathway in an epigenetic manner.

#### 3.5.6. Colchicine and Its Role against NLRP3 Inflammasome

Colchicine is a low-cost indicator drug for treating familial Mediterranean fever and gouty arthritis. Colchicine may interfere with the phlogistic processes as it blocks the production of leukotriene B4, TNF-α, TxA2, prostaglandin E2, and prevents the activity of COX 2 [108]. In addition, even at low concentrations exerts an anti-inflammatory action as it hinders the migration of neutrophils, preventing their adhesion with the endothelial P and the E-selectin. Several mechanisms through which colchicine may suppress the activation of NLRP3 inflammasome have been described in the literature. Novel research has demonstrated prevention of the assembly of the NLRP3 inflammasome by inhibiting the expression of pirin MEFV [109]. A recent study by Martinez et al. illustrated a new mechanism of action of colchicine characterized by the inhibition of caspase-1 monocyte in patients with acute coronary syndrome. These data have been corroborated by a further experiment performed on a mouse model with small intestine damage in which both the expression of caspase-1 and the mature form of IL-1β are inhibited by colchicine [110]. In addition, colchicine prevents P2X7-induced pore formation, resulting in a significant reduction in both ROS and IL-1β levels [111].

#### 3.5.7. Other Anti-Inflammasome Candidate Drugs

It is possible to reduce NLRP3 levels and prevent the release of cytokine IL-1β into the extracellular environment thanks to pannexin 1 inhibitor, probenecid, which causes astrocyte death and stimulates ROS production [112]. It is likely that it is plausible to reduce the extent of ischemic damage in terms of heart attack volume and cerebral oedema and hinder cognitive decay by employing numerous natural compounds such as sinomenine (a kind of alkaloid), paeoniflorin, and resveratrol. However, with little evidence in the literature, further studies on the efficacy and safety of these substances should be conducted [113,114].

Resveratrol is able to exert a neuroprotective effect following a cerebral ischemia thanks to the inhibition of the pathway TXNIP-NLRP3-IL-1β. Curcumin also inhibits the axis mentioned above by significantly reducing endoplasmic reticulum stress, safeguarding hippocampal neurons from the deleterious effects of exotoxicity of cerebral ischemia such as oedema and neurological impairment [115]. Furthermore, a dimensional reduction of the ischemic area and an attenuation of neurological disability can be achieved by suppressing Bruton’s tyrosine kinase (BTK), a crucial protein component of NLRP3 inflammasome [116]. In addition, one study elucidated the role played by minocycline, a tetracycline antibiotic, in inhibiting NLRP3 inflammasome, thus preventing the activation of microglia and thus containing ischemic damage [117]. Furthermore, a wide-spectrum serine protease inhibitor called nafamostat mesilate has been shown to prevent in MCAO and OGD ischemic models the NF-kB mediated activation of the NLRP3 inflammasome, exerting anti-inflammatory effects [118]. Additionally, necrostatin-1, downregulating the axis receptor-interacting protein (RIP)1-RIP3-dynamin-related protein (DRP)1, inhibits inflammasome activation by limiting the severity of brain damage in murine models following a cerebral hemorrhage [119].

Another molecule, bright blue G, which acts as a P2X7 receptor antagonist, has been shown to attenuate the activation of NLRP3 inflammasome in cases of hemorrhagic stroke [120].

In a table (Table 2), we outline several compounds that have a suppressive effect against the activation of NLRP3 inflammasome and, therefore, can represent the protagonists of the new frontiers of pharmacological therapy of ischemic stroke.

#### 3.5.8. Future Perspectives

It seems more advantageous to identify therapeutic strategies aimed at upmodulating or downmodulating the NLRP3 inflammasome pathway at several levels than intervening on other physiopathological mechanisms characteristic of ischemic stroke. Nevertheless, it is necessarily better to characterize many aspects of these potential therapeutic weapons. First of all, it is desirable to deepen the several patterns of expression of activation of the inflammasome mentioned above on the occasion of ischemic damage in the various brain regions and in the different cytotypes of the brain [105]. Secondarily, the frequency, severity, and duration of ischemic damage affect potential beneficial or existent effects of inflammation. Moreover, early activation of the immune system could exacerbate ischemic damage, while a late response could help mitigate ischemic injury. In light of these considerations, it may be promising to inhibit the NLRP3 inflammasome pathway at the initial stage of ischemic injury. Nonetheless, experiments conducted on animal models have highlighted a neuroprotective role by anti-neuroinflammation agents up to a week after ischemic stroke, supporting the potential benefits of long-term immunotherapy [117]. Another study on intermittent fasting showed that up to 4 months from cerebral ischemic damage, the activity of NLRP3 inflammasome was inhibited [66]. Therefore, further studies must clarify the most advantageous timing of administration of NLRP3 inhibitor with the purpose of optimizing the neuroprotective effect.

Moreover, although it is promising to design new therapeutic agents that inhibit the NLRP3 inflammasome to achieve a neuroprotective effect, some considerations need to be made. Being inflammasome one of the leading actors of innate immunity responsible for the host’s response to the insult of bacterial and viral infectious agents, inhibiting it in the acute stroke phase could make the patient more exposed to DAMPs and PAMPs and thus vulnerable to infectious diseases [123]. Fortuitously, no increased incidence of infectious diseases [124] was found in the clinical studies carried out to date in which glyburide inhibiting K^+^ efflux was administered; thus preventing the maturation of caspase-1 and pro-IL-1β. Furthermore, the inhibition of inflammasome activation could have repercussions on other diseases, including diabetes, as NLRP3 inflammasome is involved in the pathogenesis of other diseases as well as in the neuroinflammation underlying ischemic stroke [125].

## 4. NLRP1 as a Target of a Possible Anti-Inflammasome Therapeutic Strategies

The NLR protein 1 (as known as NLRP1) inflammasome was the first to be studied among all members of the inflammasomes family and is peculiarly characterized by high levels of expression in cerebral tissue. It is composed of three domains, namely, NLRP1 receptor, ASC, and the caspase-1 precursor, and can elicit the inflammatory response and cell death to pyroptosis [126]. Furthermore, numerous studies conducted on different animal models have shown that NLRP1 inflammasome is a cause of cell apoptosis due to the massive release of inflammatory cytokines such as IL-1β and IL-18. Therefore the inhibition of its activation is beneficial in downregulating the inflammatory response and meliorating brain functions [34].

In view of the contribution of NLRP1 inflammasome to the pathogenesis of ischemic stroke, it could be advantageous designing a therapeutic strategy that inhibits this inflammasome. First, however, it is necessary to delineate the mechanisms that regulate the expression and activation of this molecule in the presence of a cerebral ischemic lesion.

Fann et al. [34] conducted an in vitro and in vivo experiment on brain tissue damaged by ischemic injury. They demonstrated that by pharmacologically inhibiting P38, NF-κB, ERK, or JNK signaling pathways, the expression of NLRP1 and NLRP3 is also downregulated. Consequently, also the precursors of cytokines IL-18 and IL-1β are depleted. In addition, after the administration of IVIg, the anti-apoptotic proteins Bcl-xl and Bcl-2 bind to macrophage NLRP1 receptors, inhibiting oligomerization and the subsequent activation of inflammasome [127].

Via this pharmacological approach, it is possible to prevent the activation of the inflammasome by inhibiting the activation of NF-κB and MAPK signaling pathways through an independent pathway that includes the previously mentioned anti-apoptotic proteins.

Other research has sought to explore the possible contribution of a non-coding RNA called mir-9a-5p (MicroRNA-9a-5p) in modulation of NLRP1 expression level. Mir-9a-5p is involved in several physiological metabolic pathways that concern neurite development, dendritic growth neurogenesis, angiogenesis, and differentiation of neural progenitor cells [128]. In addition, it is also responsible for pathophysiological mechanisms that occur in neurodegenerative diseases such as Huntington’s disease, Alzheimer’s disease, vascular disease, and stroke [129].

In a study conducted by Cao et al. [130] on mice subject to middle cerebral artery occlusion (MCAO) surgery were found, in this subpopulation of rats, increased levels of all three domains constituting NLRP1 inflammasome. Activation of the latter results in increased release of cleaved caspase-1 and mature forms of cytokines interleukin-18 (IL-18) and interleukin-1β (IL-1β). The same results have been observed in cells called SY-5Y subjected to oxygen-glucose deprivation (OGD). They later found that the overexpression of mir-9a-5p leads to a downregulation of NLRP1 inflammasome, thus reducing the levels of NLRP1 proteins and the cascade activation of cytokines in OGD cells and in MCAO rats. Thus, mir-9 represents a potential therapeutic treatment that can improve ischemic injury by inhibiting cytokine storm.

## 5. NLRP2 as a Target of a Possible Anti-Inflammasome Therapeutic Strategies

In the literature, NLRP3 and NLRP1 represent two members of the family of inflammasomes best investigated, and it is now undisputed the pivotal role they played in the pathogenesis of ischemic stroke. Instead, it is necessary to clarify better the levels of expression and the functions performed by NLRP2 inflammasome under both physiological and pathological conditions, such as a cerebral ischemic injury.

Sun et al. [131] have shown that NLRP2 is constitutionally expressed at the level of the CNS and is more produced by astrocytes. In addition, higher expression levels were discovered both in vitro and in vivo under ischemic stroke conditions, further confirming the contribution of NLRP2 in ischemic stroke. This hypothesis is further supported by reducing apoptosis of OGD-treated astrocytes observed at flow cytometry after silencing the NLRP2 gene. The pathogenetic mechanism by which NLRP2 could impact the pathogenesis of cerebral ischemia may be similar to that of other members of the family of inflammasomes such as NLRP1 and NLRP3, but is not yet fully defined.

In another research conducted by Cheon et al. [132] the possible role of ASK1 (apoptosis signal-regulating kinase 1), an immune-regulator and early activator involved in the mechanism of apoptosis, activation of inflammasome NLRP2 in stroke was examined. In astrocyte cell line subjected to oxygen-glucose deprivation and reperfusion (OGD/R) injury, increased inflammasome NLRP2 levels and overexpression of pro-inflammatory cytokines IL-18 and IL-1β are observed. In addition, through silencing or inhibition of ASK1, a significant reduction in the components of the aforementioned inflammasome and cytokines in both mouse models and cultured astrocytes was observed. These data support the claim that ASK1 makes an essential contribution to ischemic stroke and make it a potential therapeutic target to try to reduce the damage and disability resulting from a cerebral ischemic injury [131].

## 6. NLRC4 and Therapeutic Strategies

Inflammasome NLRC4, formerly called Ipaf, a member of the NLR family, has been extensively studied regarding the sensing role it plays in the context of bacterial infections. At the same time, it is necessary to clarify its contribution in the field of sterile inflammations, such as neuroinflammation. The latter represents the common pathogenetic substrate to a plethora of diseases affecting the central nervous system, such as multiple sclerosis (MS), Alzheimer’s disease, and stroke. In a study by Freeman et al., the authors analyzed lysophosphatidylcholine (LPC), also called lysolecithin, a major component of low-density lipoprotein that can act as a DAMP in the process of neuroinflammation. LPC is derived under physiological conditions from the hydrolysis of phosphatidylcholine via PLA2 (phospholipase A2) and accumulates under pathological conditions. In addition, it induces microglial activation and elicits the release of IL-1β through a P2X7R-independent mechanism [133].

The authors have shown that inflammasome activation occurs canonically through LPC; these findings are further supported by increased NLRC4 expression levels observed in astrocytes of mouse models in a neuroinflammation background.

Expression levels of NLRC4 have been observed at microglia where it induces the activation of caspase-1, the protagonist of the pyroptosis process during which large quantities of cytokines are released [134]. NLRC4 also contributes to pyroptosis. Recent research by Wang et al. [135] has shown that down expression of NLRC4 in the presence of a cerebral ischemic injury decreases both inflammation and pyroptosis of microglia. It has been shown that in high glucose treated hypoxia/reoxygenation (H/R) induced microglia, there is a significant decrease in NLRC4 and inflammatory cytokines involved in the mechanism of pyroptosis by Fendrr knockdown. Fendrr (LncRNA FOXF1 adjacent non-coding developmental Regulatory RNA) is a long non-coding RNA first discovered in the murine mesoderm, implicated in many pathogenetic mechanisms, including induction of apoptosis of the cerebral endothelium in case of intracerebral hypertensive haemorrhage. Fendrr binds with HERC2 protein, an E3 ubiquitin ligase with multiple structural domains, which binds to NLRC4 inflammasome. Furthermore, while HERC2 stimulates the ubiquitination of NLRC4 protein, Fendrr could restrain this process. Thereby, in the diabetic cerebral I/R model of microglia, Fendrr overexpression can be impeded by HERC2 overexpression.

These findings make Fendrr a potential target for diabetic cerebral I/R damage.

Finally, we have schematized the potential therapeutic targets of inflammasomes NLRP1, NLRP2, and NLRC4 in the following table (Table 3).

## 7. Conclusions

In conclusion, further clinical studies should be conducted that deepen the relationship between clinical signs and ischemic stroke symptomatology and the expression levels of NLRP3 inflammasomes, NLRP1, NLRP2, and NLRC4 in damaged brain tissue. Thus, acquiring more details about the metabolic pathways involving inflammasomes in brain damage can help understand stroke pathogenesis. Furthermore, the acquisition of new pathological notions would allow the identification of new, safe, and highly effective molecules against neuroinflammation [136,137,138,139,140,141]. Thereby, it would be possible to reduce the detrimental effects caused by the activation of inflammasomes and cytokines responsible for mortality and disability resulting from an ischemic stroke.

## Figures and Tables

**Table 1:** Direct and indirect inhibitors of the NLRP3 inflammasome in ischemic stroke.

Categories	Drugs or Molecules	References
Acting on gene expression products	MCC950, Bay 11-7082, NRF2, sinomenine, curcumin, minocycline	[92]
Acting on the process of gene expression	IVIG, IFN-β, ketone metabolite hydroxybutyrate, probenecid, nafamostat mesilate	[92]
Acting on gene expression processes and gene expression products	miR-223, miR-155, resveratrol	[92]

IFN-β, Interferon-β, IVIG, intravenous immune globulin, NRF2, Nuclear factor erythroid 2-related factor 2, mir-155, MicroRNA-155, mir-223, MicroRNA-223.

**Table 2:** Cell categories of drugs directly and indirectly acting on and related effect on NLRP3 pathways after ischemic stroke.

Drug	Characteristic Features	Therapeutical Actions	References
Small molecules SB 203580 Bay-11-7082 U-0126 JNK Inhibitor V	→P38-MAPK inhibitor →NF-κB inhibitor →ERK inhibitor →JNK inhibitor	Neuroprotection during induced cerebral ischemia in mouse tMCAO model and primary neuron OGD reduced expression and activation of the inflammasome and decreased release of cytokines IL-18 and IL 1β	[34]
Glyburide	NLRP3 oligomerization inhibitor	In PC12 cell OGD, anti-inflammatory and anti-oxidative stress action	[121]
MCC950	NLRP3 oligomerization inhibitor	In photothrombotic ischemia mice and primary neuron OGD, hampered platelet activation/aggregation and thrombogenesis in vitro Alleviated neuronal cell apoptosis, reduced area size of cerebral ischemia, and neurological disability	[63,94,95]
β-hydroxybutyrate (BHB)	Kefflux inhibitor and ASC oligomerization inhibitor	Inhibited NLRP3 inflammasome priming process	[25]
Nuclear factor erythroid-2 related factor 2 (NRF2)	Redox-sensitive transcription factor	Suppressed ROS- and NF-kB, modulated TXNIP complex	[96]
Nitric oxide (NO)	Gas molecule	Suppressed ASC pyroptosome formation, caspase-1 and IL-1b release	[99]
IFN-α and IFN-β	Nonspecific NLRP3 inflammasome	Promoting phosphorylation of STAT1, inducting IL-10 production	[25]
Micro RNAs	Non-protein-coding RNA	Inhibited NLRP3 protein expression	[107]
Colchicine	Alkaloid	Prevents P2X7-induced pore formation and inhibits caspase-1	[108]
Probenecid	Pannexin 1 inhibitor	In primary astrocyte OGD, reduced expression levels of NLRP3 and caspase-1 and prevented the extracellular release of IL-1β death of astrocytes and increased production of ROS	[112]
Sinomenine	Natural alkaloid compound	In mouse tMCAO model and primary mixed glial cell OGD inhibited the release of NLRP3, ASC, cleaved caspase-1, and pro-inflammatory cytokines attenuation of cerebral oedema, neurological deficit, apoptosis of neurons and reduction of infarction activation of the AMPK pathway-mitigated activation of microglia and astrocytes following ischemic damage	[114]
Paeoniflorin	Natural bioactive monoterpene glucoside	In hippocampal slices, OGD diminished expression levels of NLRP3 and its downstream proteins safeguarded neuronal cell death	[122]
Resveratrol	Natural polyphenolic compound	In mouse endothelin-1-induced MCAO model, counteracted the activation of NLRP3 and the release of IL-1β and prevented the expression of TXNIP, promoting the reduction of cerebral oedema and the size of the infarcted area	[113,115]
Curcumin	Polyphenolic compound	Inhibited endoplasmic reticulum stress, suppressed TXNIP/ NLRP3 inflammasome stimulation	[115]
Ibrutinib (PCI-32765)	Bruton’s tyrosine kinase inhibitors	Decreased levels of IL-1β IL-6, IL-23A and infiltrating microglia	[116]
Minocycline	Antibiotic immunosuppressor	In mouse tMCAO model and BV2 cell OGD inhibited activation of microglia and signals 1 and 2 of NLRP3 inflammasome activation	[117]
Nafamostat mesilate	Synthetic serine protease inhibitor	In mouse tMCAO model and primary microglial culture, OGD altered expression profiles of inflammation mediators and induced expression of anti-inflammatory mediators	[118]
Necrostatin-1	Inhibitor of RIP1 kinase	inhibits inflammasome activation in murine models	[119]
Brilliant Blue G	P2X7 receptor antagonist	Attenuated caspase-3 dependent neuronal apoptosis	[120]

ASC, Apoptosis-associated speck-like protein containing a caspase-recruitment domain; BHB, β-hydroxybutyrate; IFN-β, interferon-β; IL-1β, interleukin-1β; JNK, Jun N-terminal Kinase; NF-κB, nuclear factor kappa B; NLRP3, NLR family pyrin domain containing 3; NO, nitric oxide; OGD/R, oxygen glucose deprivation/reperfusion; P2X7, P2X purinoceptor 7; RIP, Receptor interacting protein; ROS, reactive oxygen species; STAT1, signal transducers and activators of transcription 1; tMCAO, transient middle cerebral artery occlusion; TXNIP, thioredoxin-interaction protein.

**Table 3:** Cell categories of drugs acting on and related effect/pathways after ischemic stroke.

Drug	Characteristic Features	Therapeutical Actions	References
NLRP1 SB 203580 Bay-11-7082 U-0126 JNK Inhibitor V and SP600125 IVIg	→P38-MAPK inhibitor →NF-κB inhibitor →ERK inhibitor →JNK inhibitor Intravenous immune globuline	Reduced expression levels of cleaved XIAP, cleaved caspase-1, and caspase-11 and maturation of IL-1β and IL-18	[34]
Mir-9a-5p	Non coding RNA	In OGD cells and in MCAO rats the overexpression of mir-9a-5p downregulates NLRP1 inflammasome	[130]
NLRP2 ASK-1 (Apoptosis signal-regulating kinase 1)	Immune-regulator and early activator of apoptosis	Silencing or inhibition of ASK-1 determines downexpression of NLRP2 levels	[132]
NLRC4 Fendrr	Long non-coding RNA	Fendrr knockdown in (H/R)-induced microglia reduced NLRC4 levels associated with pyroptosis	[135]

ASK-1, Apoptosis signal-regulating kinase 1; IVIg, Intravenous immune globuline; JNK, Jun N-terminal Kinase; NLRC4, NLR Family CARD-Domain-Containing 4; NLRP1, NLR family pyrin-domain-containing 1; NLRP2, NLR family pyrin-domain-containing 2.

## Data Availability

Not applicable.

## Abbreviations

2APB	2-aminoethoxy diphenylborinate
ADP	Adenosine diphosphate
ASC	Apoptosis-associated speck-like protein containing a caspase-recruitment domain
ASICs	Acid-sensing *ion* channels
ASK1	Apoptosis signal-regulating kinase 1
ATP	Adenosine triphosphate
BBB	Blood brain barrier
BHB	Beta-hydroxybutyrate
BTK	Bruton’s tyrosine kinase
CARD	Caspase activation and recruitment domain
CASr	Calcium-sensing receptor
CIAS-1	Cold-induced autoinflammatory syndrome 1
CNS	Central nervous system
CPT1A	Carnitine palmitoyltransferase 1A
DAMPs	Damage-associated molecular patterns
ER	Endoplasmic reticulum
ERK	Extracellular signal-regulated kinases
FADD	Fas-associated protein with death domain
Fendrr	LncRNA FOXF1 adjacent non-coding developmental Regulatory RNA
GPRC6A	G protein-coupled receptor family C group 6 member A
GSDMD	Gasdermin D
HERC2	HECT And RLD Domain Containing E3 Ubiquitin Protein Ligase 2
IFN	Interferon
IFNAR	Interferon-α/β receptor
IL-18	Interleukin-18
IL-1β	Interleukin-1 β
IP3	Inositol 1,4,5-triphosphate
IP3R	IP3 receptor
JAK	Janus kinase
JNK	c-Jun N-terminal kinases
LPC	Lysophosphatidylcholine
LPS	Lipopolysaccharide
LRR	Leucine-rich repeat
MAM	Mitochondria-associated membrane
MAPK	Mitogen-activated protein kinase
MAVS	Mitochondrial antiviral-signaling protein
MCC950	1-(1,2,3,5,6,7-Hexahydro-s-indacen-4-yl)-3-[4-(2-hydroxypropan-2-yl)furan-2-yl]sulfonylurea
MCP-1/CCL2	Monocyte Chemoattractant Protein-1
MMPs	Matrix metalloproteinases
MSU	Monosodium urate
mtROS	Mitochondrial ROS
Myd88	Myeloid differentiation primary response 88
NADPH	Nicotinamide adenine dinucleotide phosphate
NF-κB	Nuclear factor kappa-light-chain-enhancer of activated B cells
NLR	NOD-like receptor
NLRC4	NLR Family CARD-Domain-Containing 4
NLRP1	NLR family pyrin-domain-containing 1
NLRP2	NLR family pyrin-domain containing 2
NLRP3	NLR family pyrin-domain-containing 3
NMDA	N methyl D aspartate
NO	Nitric oxide
NOD	Nucleotide-binding oligomerization domain
NOX2	NADPH oxidase 2
NOX4	NADPH oxidase 4
NRF2	Nuclear factor erythroid-2 related factor 2
NVU	Neurovascular unit
OGD	Oxygen-glucose deprivation
P2X7R	P2X purinoceptor 7
PAMPs	Pathogen Associated Molecular Patterns
PIP2	Phosphatidylinositol 4,5-bisphosphate
PLA2	Phospholipase A2
PRRs	Pattern recognition receptors
PYD	Pyrin domain
PYHIN	Pyrin and HIN domain
RAGE	Receptor for advanced glycation end-products
ROS	Reactive oxygen species
RIPK1	Receptor-interacting serine/threonine-protein kinase 1
STAT1	Signal transducer and activator of transcription 1
STAT3	Signal transducer and activator of transcription 3
TAK1	Transforming growth factor beta-activated kinase 1
tMCAO	Transient middle cerebral artery occlusion
TLRs	Toll-like receptors
TRAF3	TNF-receptor-associated factor 3
TRAF6	TNF-receptor-associated factor 6
TRPM	Transient receptor potential ion melastatin
TRPV	Transient receptor potential ion vanilloid
TXNIP	Thioredoxin-interacting protein
VDAC	Voltage-dependent anion channel
